# High-resolution magnetic resonance imaging findings of basilar artery plaque in a patient with branch atheromatous disease: a case report

**DOI:** 10.1186/1752-1947-8-395

**Published:** 2014-11-29

**Authors:** Yosuke Miyaji, Yuichi Kawabata, Hideto Joki, Shunsuke Seki, Kentaro Mori, Tomoya Kamide, Akira Tamase, Motohiro Nomura, Yoshihisa Kitamura, Fumiaki Tanaka

**Affiliations:** 8 Department of Neurology and Stroke Medicine, Yokohama Sakae Kyosai Hospital, 132 Katsura-cho, Sakae-ku, Yokohama, 247-8581 Japan; 9 Department of Neurosurgery and Stroke Medicine, Yokohama Sakae Kyosai Hospital, 132 Katsura-cho, Sakae-ku, Yokohama, 247-8581 Japan; 10 Department of Neurology and Stroke Medicine, Yokohama City University Graduate School of Medicine, 3-9 Fukuura, Kanazawa-ku, Yokohama, 236-0004 Japan

**Keywords:** Basilar artery, Branch atheromatous disease, Magnetic resonance imaging, Paramedian pontine artery, Progressive motor deficits

## Abstract

**Introduction:**

Intracranial branch atheromatous disease is a type of ischemic stroke that is caused by narrowing or occlusion of the orifice of the penetrating artery by atheromatous plaque. Pontine branch atheromatous disease is usually diagnosed using indirect findings such as the extension of a lesion to the basal surface of the pons because of the difficulty of demonstrating plaque in the basilar artery.

**Case presentation:**

A 72-year-old Japanese man developed sudden dysarthria and left hemiparesis, and his symptoms deteriorated thereafter. Brain magnetic resonance imaging revealed an acute infarction in the territory of the right paramedian pontine artery extending to the basal surface. Non-contrast-enhanced three-dimensional fast spin-echo T1 imaging with variable flip angles and three-dimensional fast imaging with steady-state acquisition revealed a plaque in the dorsal wall of the basilar artery that spread to the origin of the paramedian pontine artery that branched toward the infarction. Although antithrombotic agents were started, the left hemiparesis got worse and became flaccid on the following day.

**Conclusions:**

This is the first report to confirm the pathological basis of branch atheromatous disease by three-dimensional images using the new modalities of 3-Tesla magnetic resonance imaging. The use of these techniques will foster better understanding of the clinicopathological mechanisms of branch atheromatous disease.

## Introduction

Intracranial branch atheromatous disease (BAD) is a type of ischemic stroke that is caused by narrowing or occlusion of the orifice of the penetrating artery by atheromatous plaque. Pontine BAD is usually diagnosed using indirect magnetic resonance imaging (MRI) findings such as the extension of a lesion to the basal surface of the pons. Here we report a case of pontine BAD diagnosed directly by the radiographic demonstration of BAD pathophysiology using 3-Tesla MRI.

## Case presentation

**Figure 1:**
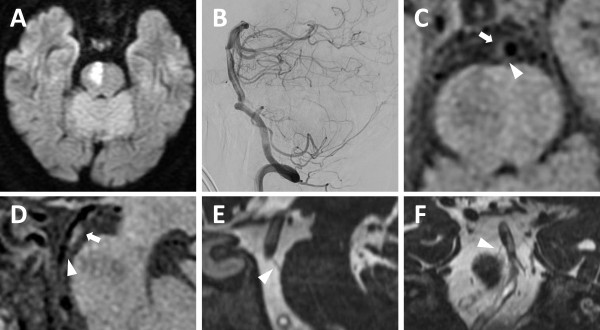
**Magnetic resonance imaging. (A)** Axial diffusion-weighted image showing an acute infarction in the territory of the right paramedian pontine artery that extends to the basal surface. **(B)** Sagittal digital subtraction angiography image showing moderate stenosis (<50%) in the basilar artery. Axial **(C)** and sagittal **(D)** non-contrast-enhanced three-dimensional fast spin-echo T1 imaging with variable flip angles (Cube T1) showing a plaque in the dorsal wall of the basilar artery (arrow). The orifice of the paramedian pontine artery is shown by the arrowhead. Sagittal **(E)** and coronal **(F)** three-dimensional fast imaging employing steady-state acquisition (3D-FIESTA) showing the right paramedian pontine artery (arrowhead). The ischemic lesion appears as a hypointense signal on the Cube T1 **(C and D)** and a hyperintense signal on the 3D-FIESTA **(E)**.

A 72-year-old Japanese man with hypertension, diabetes mellitus, and dyslipidemia presented with sudden left hemiparesis in the early afternoon. He developed dysarthria and choked on food and drink in the evening. By the next morning, the left hemiparesis had progressed and he could not stand up. He was taken by ambulance and admitted to our hospital; on admission a neurological examination revealed dysarthria and left hemiparesis involving his face, arm, and leg. MRI (Discovery MR750w 3.0T, GE Medical Systems, Milwaukee, WI, USA) revealed an acute infarction in the territory of the right paramedian pontine artery (PPA) extending to the basal surface (Figure 1A). MR angiography showed only a slightly irregular wall of the basilar artery. Digital subtraction angiography showed moderate stenosis of less than 50% in the basilar artery (Figure 1B). MRI was reexamined and a plaque was identified in the dorsal wall of the basilar artery that spread to the origin of the PPA, which branched toward the infarction. This plaque was evident on images obtained with a non-contrast-enhanced three-dimensional fast spin-echo T1 imaging with variable flip angles (Cube T1) sequence (Figure 1C-1D) and images obtained with a three-dimensional fast imaging with steady-state acquisition (3D-FIESTA) sequence (Figure 1E-1F). Although antithrombotic agents were started, his left hemiparesis got worse and became flaccid on the following day. On the 25th day, he was transferred to the rehabilitation hospital.

## Discussion

Caplan described BAD as cerebral infarction caused by narrowing or occlusion of the mouth of the branching artery by an atheromatous process that is different from lipohyalinosis causing lacunar infarction [1]. Recently, an autopsy case was reported that supports these histopathological characteristics [2]. Because of the difficulty in demonstrating plaque in the basilar artery on conventional MRI, pontine BAD has been diagnosed indirectly by the extension of a lesion to the basal surface of the pons. However, accurate radiological diagnosis based on arterial pathology is necessary as BAD has been associated with progressive motor deficits and poor prognosis in comparison with lacunar infarction [3]. Although high-resolution MRI has successfully evaluated plaque in the major artery in small, deep infarction [4], it did not illustrate the position of the plaque in relation to the penetrating artery.

Cube T1 is a volumetric imaging technique with isotropic voxels that enables reformation of black-blood images into any plane and allows detection of atherosclerotic plaques with high resolution. In addition, we depicted the outer contour of the PPA using 3D-FIESTA, which detects small amounts of fluid based on the long T2 relaxation. These new reconstructive modalities of MRI clearly and three-dimensionally demonstrated the presence of atherosclerotic plaque in the major artery at the origin of the penetrating artery, which is the pathological basis of BAD.

## Conclusions

We reported the case of a patient with a typical clinical course of BAD and demonstrated the pathological basis of BAD using 3-Tesla Cube T1 and 3D-FIESTA MRI techniques. At present, the recognition of BAD is insufficient and the use of these high-resolution MRI modalities will foster better understanding of the clinicopathological mechanisms of BAD.

## Consent

Written informed consent was obtained from the patient for publication of this case report and accompanying images. A copy of the written consent is available for review by the Editor-in-Chief of this journal.

## References

[1] Caplan LR: **Intracranial branch atheromatous disease: a neglected, understudied, and underused concept.** *Neurology* 1989, **39:**1246–1250. 10.1212/WNL.39.9.1246 10.1212/wnl.39.9.1246 2671793

[2] Tatsumi S, Yamamoto T: **An autopsied case of an apparent pontine branch atheromatous disease.** *Eur Neurol* 2010, **63:**184–185. 10.1159/000290248 10.1159/000290248 20203514

[3] Yamamoto Y, Ohara T, Hamanaka M, Hosomi A, Tamura A, Akiguchi I: **Characteristics of intracranial branch atheromatous disease and its association with progressive motor deficits.** *J Neurol Sci* 2011, **304:**78–82. 10.1016/j.jns.2011.02.006 10.1016/j.jns.2011.02.006 21402390

[4] Chung JW, Kim BJ, Sohn CH, Yoon BW, Lee SH: **Branch atheromatous plaque: a major cause of lacunar infarction (high-resolution MRI study).** *Cerebrovasc Dis Extra* 2012, **2:**36–44. 10.1159/000341399 10.1159/000341399 PMC3468813 23060895

